# Crystal structure of diethyl 2-[(2-sulfan­yl­quinolin-3-yl)methyl­idene]malonate

**DOI:** 10.1107/S2056989015013596

**Published:** 2015-07-22

**Authors:** Rihanabanu, B. R. Anitha, T. G. Meenakshi, K. Mahesh Kumar, H. C. Devarajegowda

**Affiliations:** a Department of Physics, Govt. First Grade College, Davangere 577 004, Karnataka, India; b Department of Physics, Yuvaraja’s College (Constituent College), University of Mysore, Mysore 570 005, Karnataka, India; c Department of Physics, Y. Y. D. Govt. First Grade College, Belur 573 115, Hassan, Karnataka, India; d Department of Chemistry, Karnatak University’s Karnatak Science College, Dharwad, Karnataka 580 001, India

**Keywords:** crystal structure, diester, quinoline, malonate, inter­molecular inter­actions

## Abstract

In the title compound, C_17_H_17_N O_4_S, the quinoline ring system is nearly planar, with a maximum deviation of 0.0496 (16) Å. A weak intra­molecular C—H⋯O inter­action is observed. In the crystal, C—H⋯O, S—H⋯N and π–π stacking inter­actions between the fused benzene ring of quinoline and the pyridine moieties [shortest centroid–centroid distance = 3.6754 (11) Å] are observed. Inversion-related weak C—H⋯O inter­molecular inter­actions diagonally along [010], with *R*_2_^2^(10) ring motifs, and S—H⋯N inter­molecular inter­actions diagonally along [100], with *R*_2_^2^(8) ring motifs, are present, forming a three-dimensional network structure. No classical hydrogen bonds are observed.

## Related literature   

For biological applications of quinolines, see: Nandeshwarappa *et al.*(2006 ▸); Noda *et al.* (2001 ▸); Pandey *et al.* (2004 ▸); Sharma *et al.* (2008 ▸).
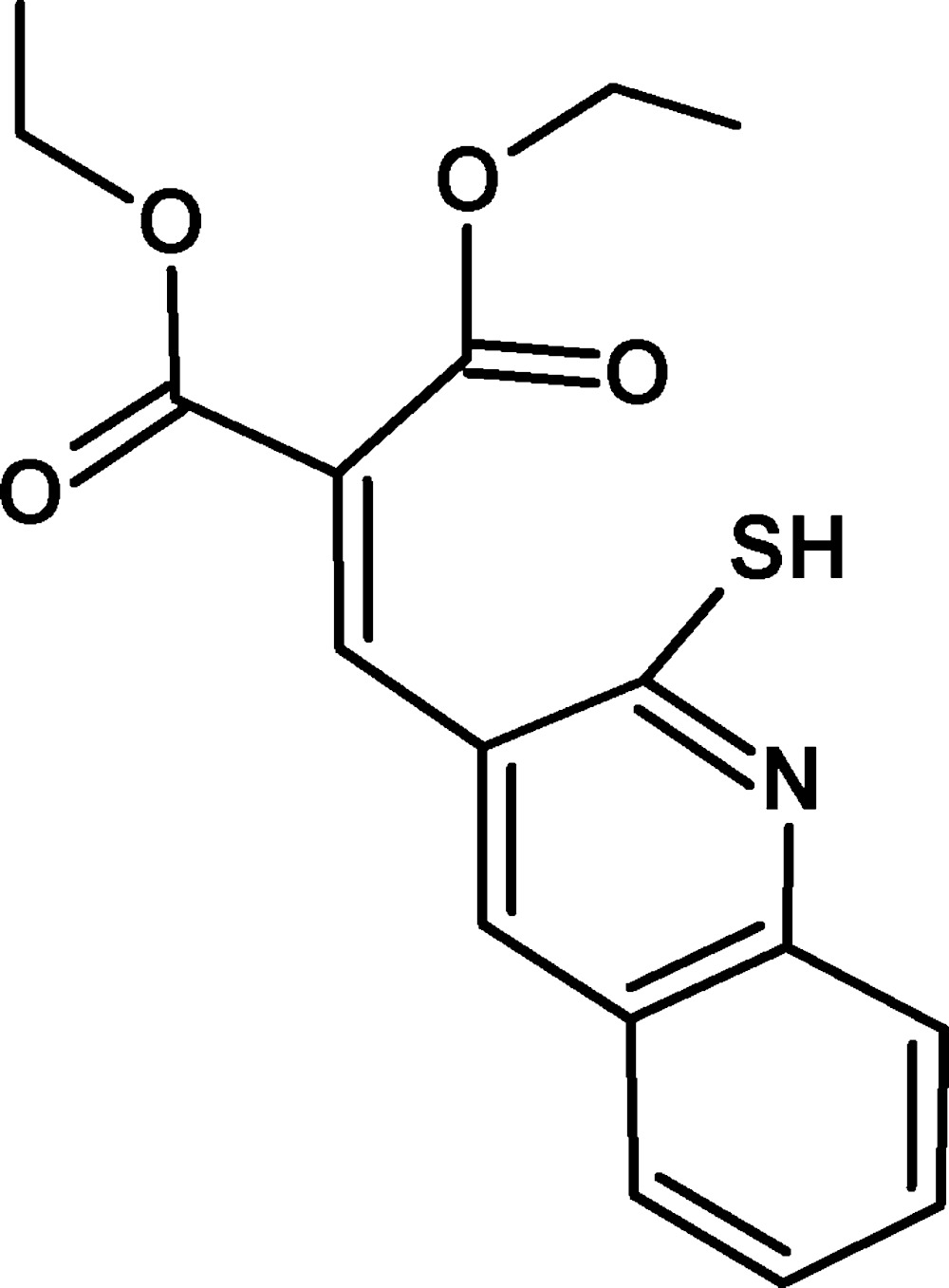


## Experimental   

### Crystal data  

C_17_H_17_NO_4_S
*M* *_r_* = 331.37
Triclinic, 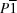

*a* = 7.3739 (4) Å
*b* = 7.8148 (4) Å
*c* = 15.8149 (7) Å
α = 90.158 (2)°
β = 99.486 (2)°
γ = 113.301 (2)°
*V* = 823.24 (7) Å^3^
*Z* = 2
Mo *K*α radiation
μ = 0.22 mm^−1^
*T* = 296 K
0.24 × 0.20 × 0.12 mm

### Data collection  

Bruker SMART CCD area-detector diffractometer
Absorption correction: multi-scan (*SADABS*; Sheldrick, 2007 ▸) *T*_min_ = 0.770, *T*_max_ = 1.000
21213 measured reflections
5853 independent reflections
4295 reflections with *I* > 2σ(*I*)
*R*_int_ = 0.025

### Refinement  

*R*[*F* ^2^ > 2σ(*F* ^2^)] = 0.062
*wR*(*F* ^2^) = 0.213
*S* = 1.04
5853 reflections
234 parameters
H atoms treated by a mixture of independent and constrained refinement
Δρ_max_ = 0.98 e Å^−3^
Δρ_min_ = −0.48 e Å^−3^

### 

Data collection: *SMART* (Bruker, 2001 ▸); cell refinement: *SAINT* (Bruker, 2001 ▸); data reduction: *SAINT*; program(s) used to solve structure: *SHELXS2014* (Sheldrick, 2008 ▸); program(s) used to refine structure: *SHELXL2014* (Sheldrick, 2015 ▸); molecular graphics: *ORTEP-3 for Windows* (Farrugia, 2012 ▸); software used to prepare material for publication: *SHELXL2014*.

## Supplementary Material

Crystal structure: contains datablock(s) I, global. DOI: 10.1107/S2056989015013596/jj2194sup1.cif

Structure factors: contains datablock(s) I. DOI: 10.1107/S2056989015013596/jj2194Isup2.hkl

Supporting information file. DOI: 10.1107/S2056989015013596/jj2194Isup3.cml

**Fig. 1.**
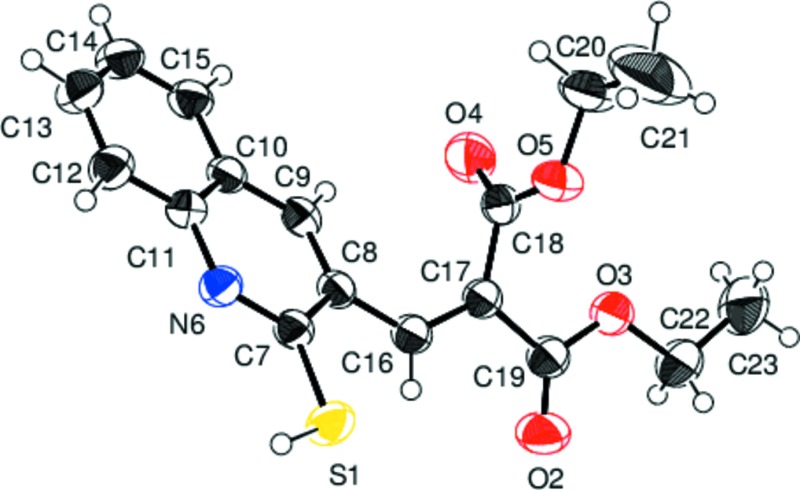
ORTEP diagram of the title compound, C17H17N O4S. Displacement ellipsoids are drawn at the 50% probability level. Hydrogen atoms are shown as spheres of arbitrary radius.

17 17 4 . DOI: 10.1107/S2056989015013596/jj2194fig1.tif
ORTEP diagram of the title compound, C_17_H_17_N O_4_S. Displacement ellipsoids are drawn at the 50% probability level. Hydrogen atoms are shown as spheres of arbitrary radius.

**Fig. 2.**
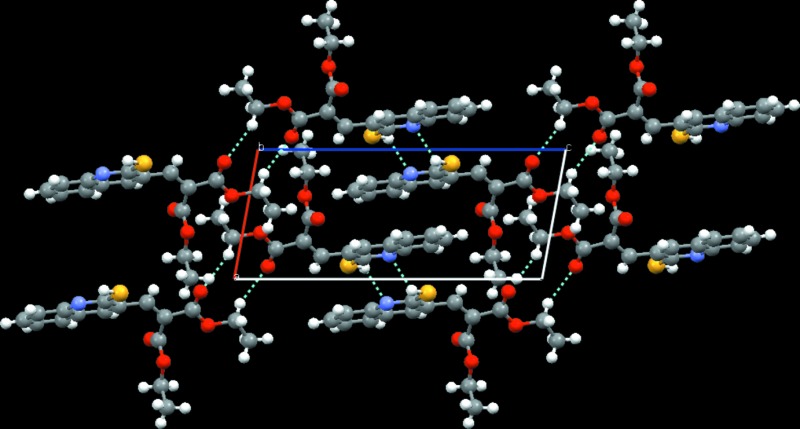
A view of the packing in the title molecule, C17H17N O4S, along the a axis. Dashed lines indicate weak C—H···O and S—H···N intermolecular interactions with inversio- related C—H···O intermolecular interactions diagonally along [010] with R22(10) ring motifs and S—H···N intermolecular interactions diagonally along [100] with R22(8) ring motifs forming a three-dimensional network structure.

Click here for additional data file.17 17 4 a . DOI: 10.1107/S2056989015013596/jj2194fig2.tif
A view of the packing in the title mol­ecule, C_17_H_17_N O_4_S, along the *a* axis. Dashed lines indicate weak C—H⋯O and S—H⋯N inter­molecular inter­actions with inversio- related C—H⋯O inter­molecular inter­actions diagonally along [010] with 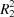
(10) ring motifs and S—H⋯N inter­molecular inter­actions diagonally along [100] with 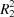
(8) ring motifs forming a three-dimensional network structure.

CCDC reference: 1413116

Additional supporting information:  crystallographic information; 3D view; checkCIF report

## Figures and Tables

**Table 1:** Hydrogen-bond geometry (, )

*D*H*A*	*D*H	H*A*	*D* *A*	*D*H*A*
S1H1N6^i^	1.20	2.37	3.3389(14)	136
C9H9O4	0.99(3)	2.41(3)	3.122(2)	129(2)
C22H22*B*O2^ii^	0.97	2.52	3.438(4)	158

Symmetry codes: (i) 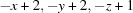; (ii) 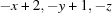.

## supplementary crystallographic information

### S1. Comment

Quinolines are a heterocyclic class of organic compounds containing a pyridine ring fused with benzene found in nature mainly in plants. Alkaloid quinine is a traditional anti-malarial drug also used in tonics. The quinoline skeleton has since been used as a basis for design of many synthetic anti-malarial compounds, of which chloroquinoline is one such example. Despite its relatively low efficacy and tolerability, quinine still plays an important role in the treatment of multi resistant malaria (Nandeshwarappa *et al.*2006). It has also played a historical role in organic chemistry as a target for structural determination and total synthesis reactions (Sharma *et al.*2008), as well as stereo selective (Noda *et al.*2001) and *enantio* selective (Pande *et al.*, 2004) total synthesis reactions. The chemistry of quinoline has gained increasing attention due to its various diverse pharmacological activities. We report herin the crystal structure of a new quinoline derivative, diethyl 2-((2-mercaptoquinolin-3-yl) methylene)malonate, C_17_H_17_N O_4_S, (I) (Fig. 1).

In the asymmetric unit of (I), the quinoline ring system is nearly planar, with a maximum deviation of 0.0496 (16) Å for atom C8. In the crystal, weak intramolecular C—H···O, intermolecular C—H···O, S—H···N (Table 1) and π–π stacking interactions between the fused benzene ring of quinoline, Cg(2) [C10—C15], and pyridine, Cg(1) [N6//C7–C11], [shortest centroid–centroid distance = 3.6751 (11) Å] are observed. Inversion related weak C—H···O intermolecular interactions diagonally along [010] with *R*_2_^2^(10) ring motifs and S—H···N intermolecular interactions diagonally along [100] with *R*_2_^2^(8) ring motifs are present forming a three-dimensional network structure (Fig. 2). No classical hydrogen bonds are observed.

### S2. Experimental

All the chemicals of analytical reagent grade were used directly without further purification. An equimolar quantity of 2-mercapto-3-formyl quinoline (0.01 mm) and diethylmalonate (0.001mm) were refluxed for 24 hr in acetonitrile at 353 K. After completion of the reaction the solvent was removed from the vacuue and recrystalized from ethanol. Yellow needles of the title compound were grown from ethanol solution by slow evaporation at room temperature. Colour: Yellow. Yield= 82%, m.p.:458 K.

### S3. Refinement

All H atoms were positioned geometrically, with S—H = 1.2 Å, C—H = 0.93 Å for aromatic H, C—H = 0.97 Å for methylene H and C—H = 0.96 Å for methyl H,and refined using a riding model with *U*_iso_(H) = 1.5*U*_eq_(C) for methyl H and *U*_iso_(H) = 1.2*U*_eq_(C) for all other H.

### Crystal data

**Table:**

C_17_H_17_NO_4_S	*F*(000) = 348
*M**_r_* = 331.37	*D*_x_ = 1.337 Mg m^−^^3^
Triclinic, *P*1	Melting point: 458 K
*a* = 7.3739 (4) Å	Mo *K*α radiation, λ = 0.71073 Å
*b* = 7.8148 (4) Å	Cell parameters from 5853 reflections
*c* = 15.8149 (7) Å	θ = 2.6–32.5°
α = 90.158 (2)°	µ = 0.22 mm^−^^1^
β = 99.486 (2)°	*T* = 296 K
γ = 113.301 (2)°	Plate, yellow
*V* = 823.24 (7) Å^3^	0.24 × 0.20 × 0.12 mm
*Z* = 2	

### Data collection

**Table:**

Bruker SMART CCD area-detector diffractometer	5853 independent reflections
Radiation source: fine-focus sealed tube	4295 reflections with *I* > 2σ(*I*)
Graphite monochromator	*R*_int_ = 0.025
Detector resolution: 10.0 pixels mm^-1^	θ_max_ = 32.5°, θ_min_ = 2.6°
ω and φ scans	*h* = −11→10
Absorption correction: multi-scan (*SADABS*; Sheldrick, 2007)	*k* = −11→11
*T*_min_ = 0.770, *T*_max_ = 1.000	*l* = −23→23
21213 measured reflections	

### Refinement

**Table:**

Refinement on *F*^2^	0 restraints
Least-squares matrix: full	Hydrogen site location: inferred from neighbouring sites
*R*[*F*^2^ > 2σ(*F*^2^)] = 0.062	H atoms treated by a mixture of independent and constrained refinement
*wR*(*F*^2^) = 0.213	*w* = 1/[σ^2^(*F*_o_^2^) + (0.1265*P*)^2^ + 0.1563*P*] where *P* = (*F*_o_^2^ + 2*F*_c_^2^)/3
*S* = 1.04	(Δ/σ)_max_ < 0.001
5853 reflections	Δρ_max_ = 0.98 e Å^−^^3^
234 parameters	Δρ_min_ = −0.48 e Å^−^^3^

### Special details

**Table:**

Geometry. All e.s.d.'s (except the e.s.d. in the dihedral angle between two l.s. planes) are estimated using the full covariance matrix. The cell e.s.d.'s are taken into account individually in the estimation of e.s.d.'s in distances, angles and torsion angles; correlations between e.s.d.'s in cell parameters are only used when they are defined by crystal symmetry. An approximate (isotropic) treatment of cell e.s.d.'s is used for estimating e.s.d.'s involving l.s. planes.

### Fractional atomic coordinates and isotropic or equivalent isotropic displacement parameters (Å^2^)

**Table:**

	*x*	*y*	*z*	*U*_iso_*/*U*_eq_	
S1	0.89170 (8)	0.93555 (6)	0.36268 (3)	0.05361 (17)	
H1	0.9130	1.0542	0.4163	0.080*	
O2	0.9018 (3)	0.6179 (2)	0.10198 (11)	0.0741 (5)	
O3	0.6426 (2)	0.3469 (2)	0.05791 (8)	0.0546 (3)	
O4	0.5360 (3)	0.1266 (2)	0.23539 (12)	0.0772 (5)	
O5	0.3614 (2)	0.2881 (2)	0.17989 (10)	0.0584 (4)	
N6	0.8126 (2)	0.71751 (18)	0.48999 (9)	0.0417 (3)	
C7	0.8326 (2)	0.7292 (2)	0.40651 (10)	0.0380 (3)	
C8	0.7963 (2)	0.5561 (2)	0.35911 (10)	0.0370 (3)	
C9	0.7551 (2)	0.3953 (2)	0.40069 (10)	0.0398 (3)	
C10	0.7342 (2)	0.3901 (2)	0.48846 (10)	0.0383 (3)	
C11	0.7609 (2)	0.5567 (2)	0.53269 (10)	0.0388 (3)	
C12	0.7363 (3)	0.5596 (3)	0.61848 (11)	0.0491 (4)	
C13	0.6855 (3)	0.3962 (3)	0.65909 (13)	0.0552 (4)	
C14	0.6621 (3)	0.2299 (3)	0.61664 (14)	0.0555 (5)	
C15	0.6872 (3)	0.2268 (2)	0.53337 (13)	0.0480 (4)	
C16	0.8203 (2)	0.5658 (2)	0.26908 (10)	0.0409 (3)	
C17	0.7083 (2)	0.4422 (2)	0.20334 (10)	0.0408 (3)	
C18	0.5287 (3)	0.2679 (2)	0.20902 (10)	0.0452 (4)	
C19	0.7642 (3)	0.4825 (3)	0.11661 (11)	0.0480 (4)	
C20	0.1759 (3)	0.1201 (4)	0.17362 (19)	0.0786 (7)	
H20A	0.1767	0.0250	0.1343	0.094*	
H20B	0.1638	0.0706	0.2296	0.094*	
C21	0.0112 (5)	0.1699 (7)	0.1429 (4)	0.153 (2)	
H21A	0.0306	0.2843	0.1731	0.229*	
H21B	−0.1114	0.0724	0.1525	0.229*	
H21C	0.0038	0.1864	0.0825	0.229*	
C22	0.6792 (4)	0.3723 (4)	−0.03002 (13)	0.0671 (6)	
H22A	0.6744	0.4891	−0.0482	0.081*	
H22B	0.8109	0.3761	−0.0333	0.081*	
C23	0.5247 (5)	0.2163 (5)	−0.08543 (16)	0.0886 (9)	
H23A	0.5372	0.1023	−0.0698	0.133*	
H23B	0.5403	0.2350	−0.1442	0.133*	
H23C	0.3946	0.2086	−0.0788	0.133*	
H9	0.737 (4)	0.276 (4)	0.3721 (18)	0.066 (7)*	
H12	0.762 (4)	0.689 (4)	0.6437 (17)	0.063 (7)*	
H13	0.658 (4)	0.395 (4)	0.715 (2)	0.079 (8)*	
H14	0.628 (5)	0.139 (5)	0.643 (2)	0.081 (9)*	
H15	0.659 (4)	0.107 (4)	0.5002 (17)	0.067 (7)*	
H16	0.917 (3)	0.662 (3)	0.2553 (14)	0.044 (5)*	

### Atomic displacement parameters (Å^2^)

**Table:**

	*U*^11^	*U*^22^	*U*^33^	*U*^12^	*U*^13^	*U*^23^
S1	0.0738 (3)	0.0359 (2)	0.0479 (3)	0.0171 (2)	0.0148 (2)	0.00743 (17)
O2	0.0679 (9)	0.0726 (10)	0.0589 (8)	−0.0024 (8)	0.0283 (7)	−0.0006 (7)
O3	0.0549 (7)	0.0603 (8)	0.0379 (6)	0.0098 (6)	0.0140 (5)	−0.0042 (5)
O4	0.0872 (12)	0.0437 (7)	0.0777 (11)	0.0108 (7)	−0.0076 (9)	0.0067 (7)
O5	0.0415 (6)	0.0578 (8)	0.0658 (8)	0.0070 (5)	0.0153 (6)	0.0071 (6)
N6	0.0510 (7)	0.0334 (6)	0.0386 (6)	0.0135 (5)	0.0112 (5)	0.0010 (5)
C7	0.0385 (7)	0.0340 (6)	0.0382 (7)	0.0113 (5)	0.0064 (5)	0.0000 (5)
C8	0.0334 (6)	0.0358 (6)	0.0370 (6)	0.0097 (5)	0.0042 (5)	−0.0023 (5)
C9	0.0377 (7)	0.0339 (6)	0.0426 (7)	0.0109 (5)	0.0021 (5)	−0.0043 (5)
C10	0.0329 (6)	0.0341 (6)	0.0424 (7)	0.0089 (5)	0.0037 (5)	0.0026 (5)
C11	0.0365 (7)	0.0365 (7)	0.0400 (7)	0.0107 (5)	0.0080 (5)	0.0040 (5)
C12	0.0539 (9)	0.0499 (9)	0.0425 (8)	0.0176 (7)	0.0146 (7)	0.0047 (7)
C13	0.0530 (10)	0.0634 (11)	0.0481 (9)	0.0188 (8)	0.0174 (8)	0.0153 (8)
C14	0.0499 (9)	0.0497 (10)	0.0597 (11)	0.0121 (7)	0.0108 (8)	0.0200 (8)
C15	0.0440 (8)	0.0370 (7)	0.0558 (9)	0.0104 (6)	0.0049 (7)	0.0077 (7)
C16	0.0379 (7)	0.0400 (7)	0.0408 (7)	0.0112 (6)	0.0082 (6)	−0.0002 (6)
C17	0.0396 (7)	0.0429 (7)	0.0370 (7)	0.0128 (6)	0.0090 (5)	0.0005 (6)
C18	0.0496 (8)	0.0419 (8)	0.0340 (7)	0.0084 (6)	0.0062 (6)	−0.0028 (6)
C19	0.0449 (8)	0.0536 (9)	0.0419 (8)	0.0142 (7)	0.0128 (6)	0.0002 (7)
C20	0.0509 (11)	0.0720 (15)	0.0844 (16)	−0.0077 (10)	0.0189 (11)	0.0015 (12)
C21	0.0525 (17)	0.125 (3)	0.240 (6)	0.0057 (18)	−0.007 (2)	0.052 (4)
C22	0.0709 (13)	0.0810 (15)	0.0411 (9)	0.0172 (11)	0.0226 (9)	0.0020 (9)
C23	0.115 (2)	0.0886 (19)	0.0448 (11)	0.0240 (17)	0.0127 (12)	−0.0065 (11)

### Geometric parameters (Å, º)

**Table:**

S1—C7	1.6796 (16)	C13—H13	0.94 (3)
S1—H1	1.2000	C14—C15	1.361 (3)
O2—C19	1.197 (2)	C14—H14	0.80 (3)
O3—C19	1.326 (2)	C15—H15	1.00 (3)
O3—C22	1.459 (2)	C16—C17	1.333 (2)
O4—C18	1.199 (2)	C16—H16	0.86 (2)
O5—C18	1.314 (2)	C17—C18	1.494 (2)
O5—C20	1.462 (3)	C17—C19	1.495 (2)
N6—C7	1.352 (2)	C20—C21	1.429 (5)
N6—C11	1.375 (2)	C20—H20A	0.9700
C7—C8	1.451 (2)	C20—H20B	0.9700
C8—C9	1.369 (2)	C21—H21A	0.9600
C8—C16	1.462 (2)	C21—H21B	0.9600
C9—C10	1.421 (2)	C21—H21C	0.9600
C9—H9	0.99 (3)	C22—C23	1.456 (4)
C10—C11	1.403 (2)	C22—H22A	0.9700
C10—C15	1.411 (2)	C22—H22B	0.9700
C11—C12	1.399 (2)	C23—H23A	0.9600
C12—C13	1.374 (3)	C23—H23B	0.9600
C12—H12	1.02 (3)	C23—H23C	0.9600
C13—C14	1.396 (3)		
			
C7—S1—H1	109.5	C16—C17—C18	125.18 (14)
C19—O3—C22	116.21 (16)	C16—C17—C19	117.59 (15)
C18—O5—C20	116.10 (19)	C18—C17—C19	117.22 (14)
C7—N6—C11	125.49 (13)	O4—C18—O5	124.31 (18)
N6—C7—C8	116.36 (14)	O4—C18—C17	124.41 (18)
N6—C7—S1	119.98 (11)	O5—C18—C17	111.27 (15)
C8—C7—S1	123.65 (12)	O2—C19—O3	124.24 (16)
C9—C8—C7	119.72 (14)	O2—C19—C17	124.71 (17)
C9—C8—C16	122.79 (14)	O3—C19—C17	111.04 (15)
C7—C8—C16	117.33 (14)	C21—C20—O5	108.0 (3)
C8—C9—C10	121.62 (14)	C21—C20—H20A	110.1
C8—C9—H9	122.7 (16)	O5—C20—H20A	110.1
C10—C9—H9	115.7 (16)	C21—C20—H20B	110.1
C11—C10—C15	118.31 (15)	O5—C20—H20B	110.1
C11—C10—C9	118.09 (14)	H20A—C20—H20B	108.4
C15—C10—C9	123.61 (15)	C20—C21—H21A	109.5
N6—C11—C12	120.63 (14)	C20—C21—H21B	109.5
N6—C11—C10	118.56 (14)	H21A—C21—H21B	109.5
C12—C11—C10	120.81 (15)	C20—C21—H21C	109.5
C13—C12—C11	118.92 (17)	H21A—C21—H21C	109.5
C13—C12—H12	127.7 (14)	H21B—C21—H21C	109.5
C11—C12—H12	113.3 (14)	C23—C22—O3	108.17 (19)
C12—C13—C14	121.07 (18)	C23—C22—H22A	110.1
C12—C13—H13	119.0 (18)	O3—C22—H22A	110.1
C14—C13—H13	119.8 (18)	C23—C22—H22B	110.1
C15—C14—C13	120.20 (17)	O3—C22—H22B	110.1
C15—C14—H14	124 (2)	H22A—C22—H22B	108.4
C13—C14—H14	116 (2)	C22—C23—H23A	109.5
C14—C15—C10	120.66 (17)	C22—C23—H23B	109.5
C14—C15—H15	121.4 (15)	H23A—C23—H23B	109.5
C10—C15—H15	117.6 (15)	C22—C23—H23C	109.5
C17—C16—C8	127.50 (15)	H23A—C23—H23C	109.5
C17—C16—H16	114.3 (14)	H23B—C23—H23C	109.5
C8—C16—H16	118.2 (14)		
			
C11—N6—C7—C8	−0.1 (2)	C11—C10—C15—C14	2.2 (2)
C11—N6—C7—S1	178.63 (13)	C9—C10—C15—C14	−177.58 (16)
N6—C7—C8—C9	−3.4 (2)	C9—C8—C16—C17	42.8 (3)
S1—C7—C8—C9	177.93 (12)	C7—C8—C16—C17	−141.69 (17)
N6—C7—C8—C16	−179.02 (14)	C8—C16—C17—C18	1.8 (3)
S1—C7—C8—C16	2.3 (2)	C8—C16—C17—C19	−179.45 (16)
C7—C8—C9—C10	4.1 (2)	C20—O5—C18—O4	−5.2 (3)
C16—C8—C9—C10	179.47 (14)	C20—O5—C18—C17	173.63 (17)
C8—C9—C10—C11	−1.3 (2)	C16—C17—C18—O4	−78.9 (3)
C8—C9—C10—C15	178.46 (15)	C19—C17—C18—O4	102.4 (2)
C7—N6—C11—C12	−177.33 (16)	C16—C17—C18—O5	102.3 (2)
C7—N6—C11—C10	2.9 (2)	C19—C17—C18—O5	−76.47 (19)
C15—C10—C11—N6	178.10 (14)	C22—O3—C19—O2	−2.2 (3)
C9—C10—C11—N6	−2.1 (2)	C22—O3—C19—C17	178.64 (18)
C15—C10—C11—C12	−1.7 (2)	C16—C17—C19—O2	−0.2 (3)
C9—C10—C11—C12	178.05 (15)	C18—C17—C19—O2	178.7 (2)
N6—C11—C12—C13	−179.72 (17)	C16—C17—C19—O3	178.96 (16)
C10—C11—C12—C13	0.1 (3)	C18—C17—C19—O3	−2.2 (2)
C11—C12—C13—C14	1.2 (3)	C18—O5—C20—C21	178.7 (3)
C12—C13—C14—C15	−0.7 (3)	C19—O3—C22—C23	−176.6 (2)
C13—C14—C15—C10	−1.0 (3)		

### Hydrogen-bond geometry (Å, º)

**Table:**

*D*—H···*A*	*D*—H	H···*A*	*D*···*A*	*D*—H···*A*
S1—H1···N6^i^	1.20	2.37	3.3389 (14)	136
C9—H9···O4	0.99 (3)	2.41 (3)	3.122 (2)	129 (2)
C22—H22*B*···O2^ii^	0.97	2.52	3.438 (4)	158

Symmetry codes: (i) −*x*+2, −*y*+2, −*z*+1; (ii) −*x*+2, −*y*+1, −*z*.
