# Supplementary figures and images for: Crystal structure of diethyl 2-[(2-sulfan­yl­quinolin-3-yl)methyl­idene]malonate
Source: Acta Crystallogr E Crystallogr Commun. 2015 Jul 22;71(Pt 8):o594–5. doi: 10.1107/S2056989015013596 (PMC4571415; doi:10.1107/S2056989015013596)

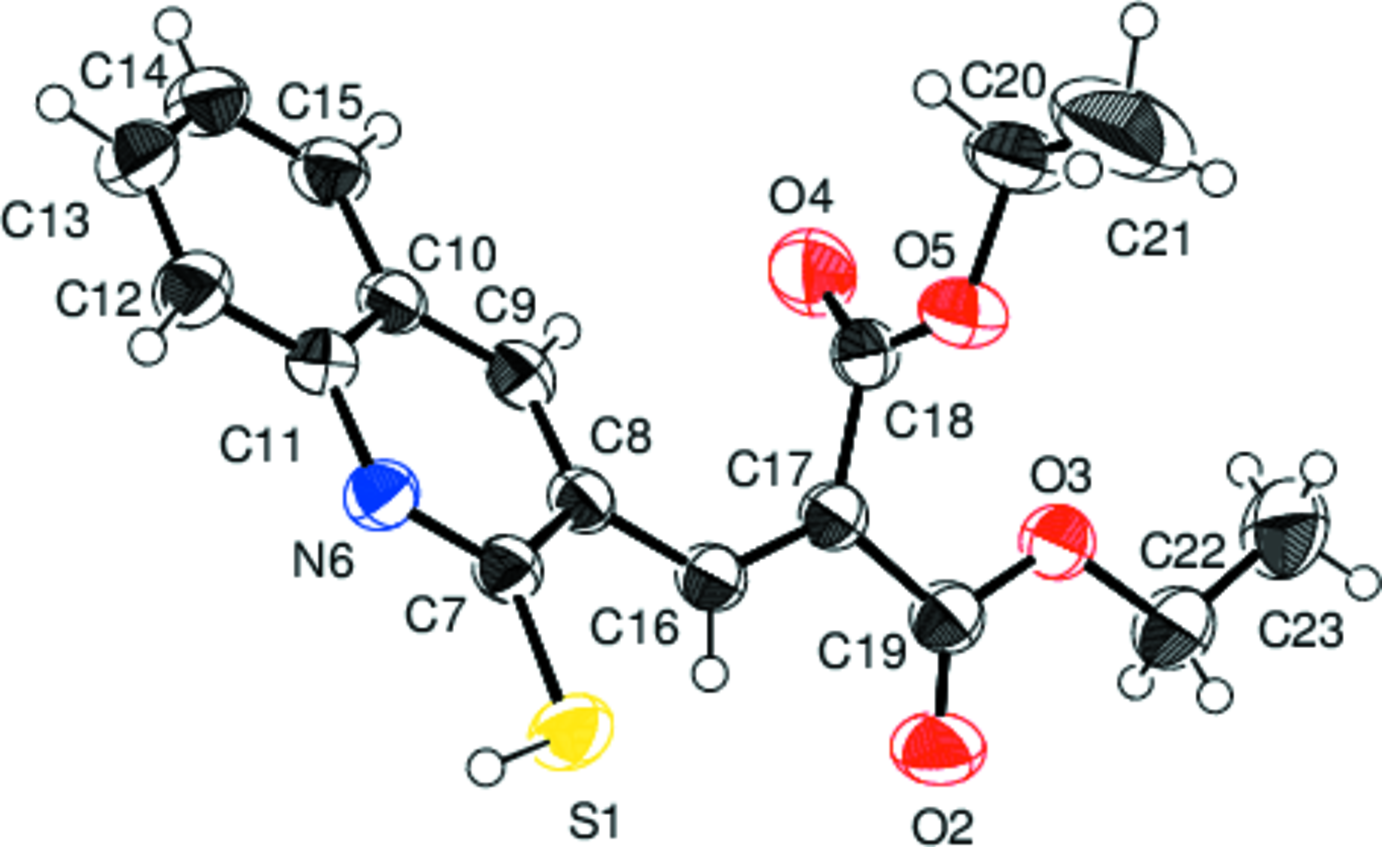

Supplement: Supplementary file 4 [file e-71-0o594-fig1.tif]

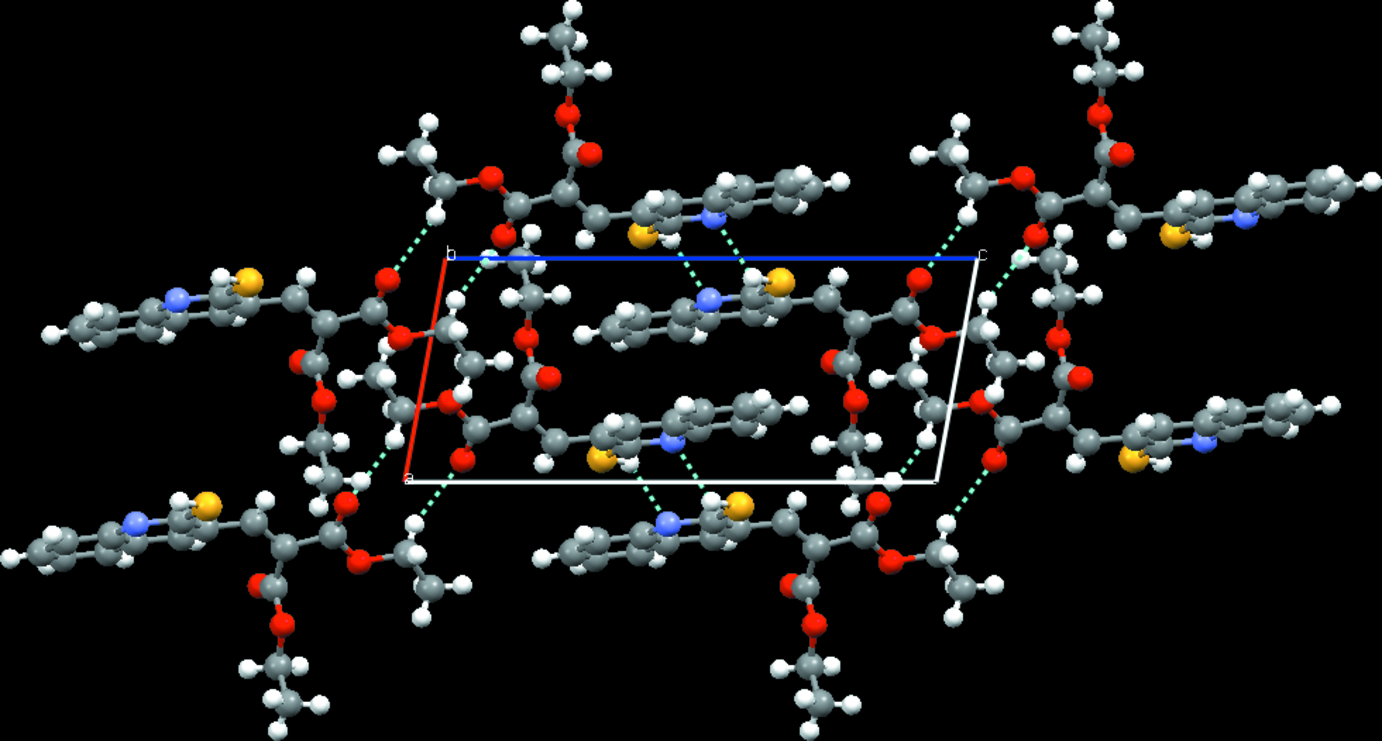

Supplement: Supplementary file 5 [file e-71-0o594-fig2.tif]
